# Supplementary material for: Sequence-only prediction of binding affinity changes: a robust and interpretable model for antibody engineering
Source: Bioinformatics. 2025 Aug 9;41(8):btaf446. doi: 10.1093/bioinformatics/btaf446 (PMC12371331; doi:10.1093/bioinformatics/btaf446)
Supplement: btaf446_Supplementary_Data [file btaf446_supplementary_data.docx]

**Supplementary Information**

**Sequence-Only Prediction of Binding Affinity Changes: A Robust and Interpretable Model for Antibody Engineering**

**Chen Liu^1^, Mingchen Li^1^, Yang Tan^1^, Wenrui Gou^1^, Guisheng Fan^1, *^,**

**and Bingxin Zhou^2, *^**

^1^School of Information Science and Engineering, East China University of Science and Technology, Shanghai, 200237, China

^2^Institute of Natural Sciences, Shanghai Jiao Tong University, shanghai, 200240, China.

*Corresponding author. bingxin.zhou@sjtu.edu.cn; gsfan@ecust.edu.cn

**
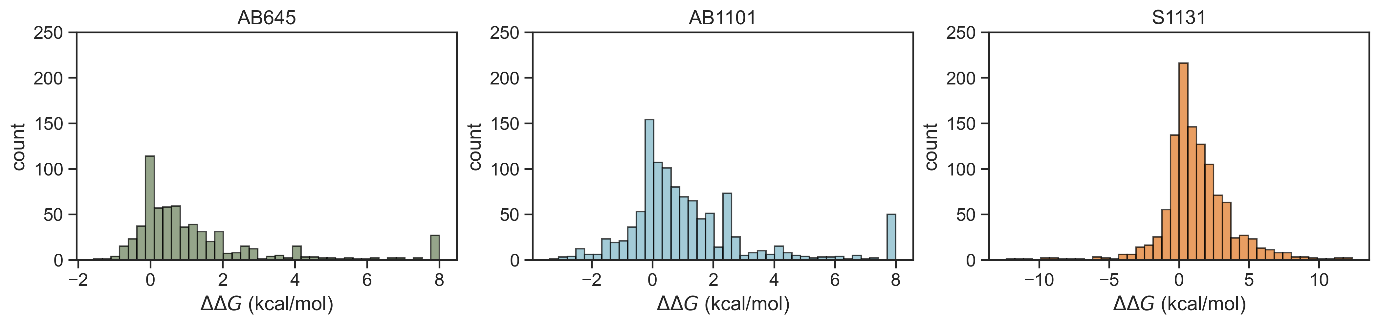
**

Fig. S1. The distribution of ∆∆G in the three benchmark datasets. Most values cluster near zero, with a higher prevalence of positive ∆∆G values, indicating that mutations generally weaken binding affinity. For AB645 and AB1101, the maximum ∆∆G values reach 8 kcal/mol, signifying variants with no detectable binding within the assay's sensitivity limits. In comparison, S1131 displays a broader, more symmetric distribution, reflecting a diverse range of mutation impacts on binding affinity and a more balanced data representation.

Table S1. The size of three datasets, including the number of mutation records and distinct proteins.

|  | AB645 | AB1101 | S1131 |
| --- | --- | --- | --- |
| Mutation records | 645 | 1101 | 1131 |
| Protein numbers | 29 | 32 | 112 |

Table S2. The different pre-trained protein language model description.

| Model | Params | Embed. Dim | Type | Checkpoint Source |
| --- | --- | --- | --- | --- |
| ESM2-650M | 650M | 1,280 | Encoder-only | <https://huggingface.co/facebook/esm2_t33_650M_UR50D> |
| ESM1b-650M | 650M | 1,280 | Encoder-only | <https://huggingface.co/facebook/esm1b_t33_650M_UR50S> |
| ProtBert | 420M | 1024 | Encoder-only | <https://huggingface.co/Rostlab/prot_bert> |
| Ankh | 450M | 768 | Encoder-Decoder | <https://huggingface.co/ElnaggarLab/ankh-base> |


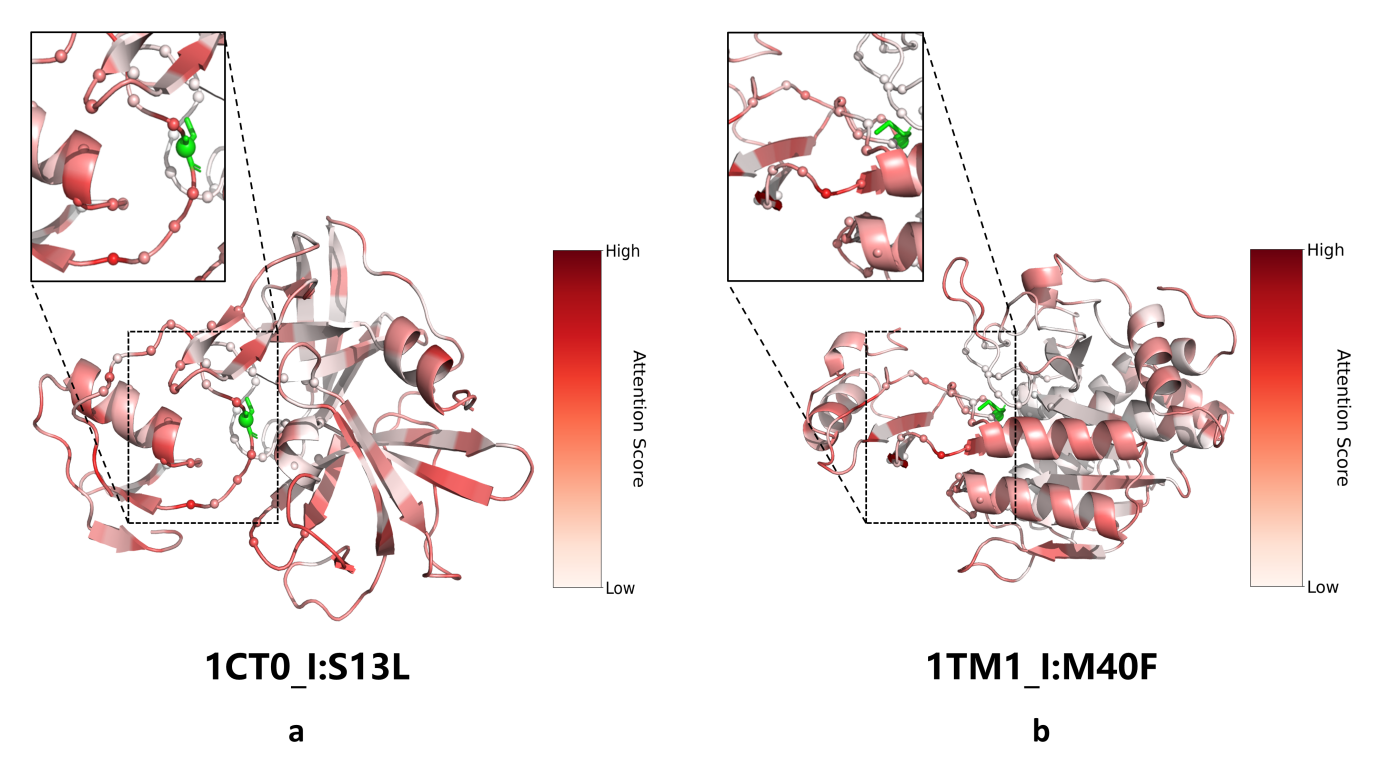


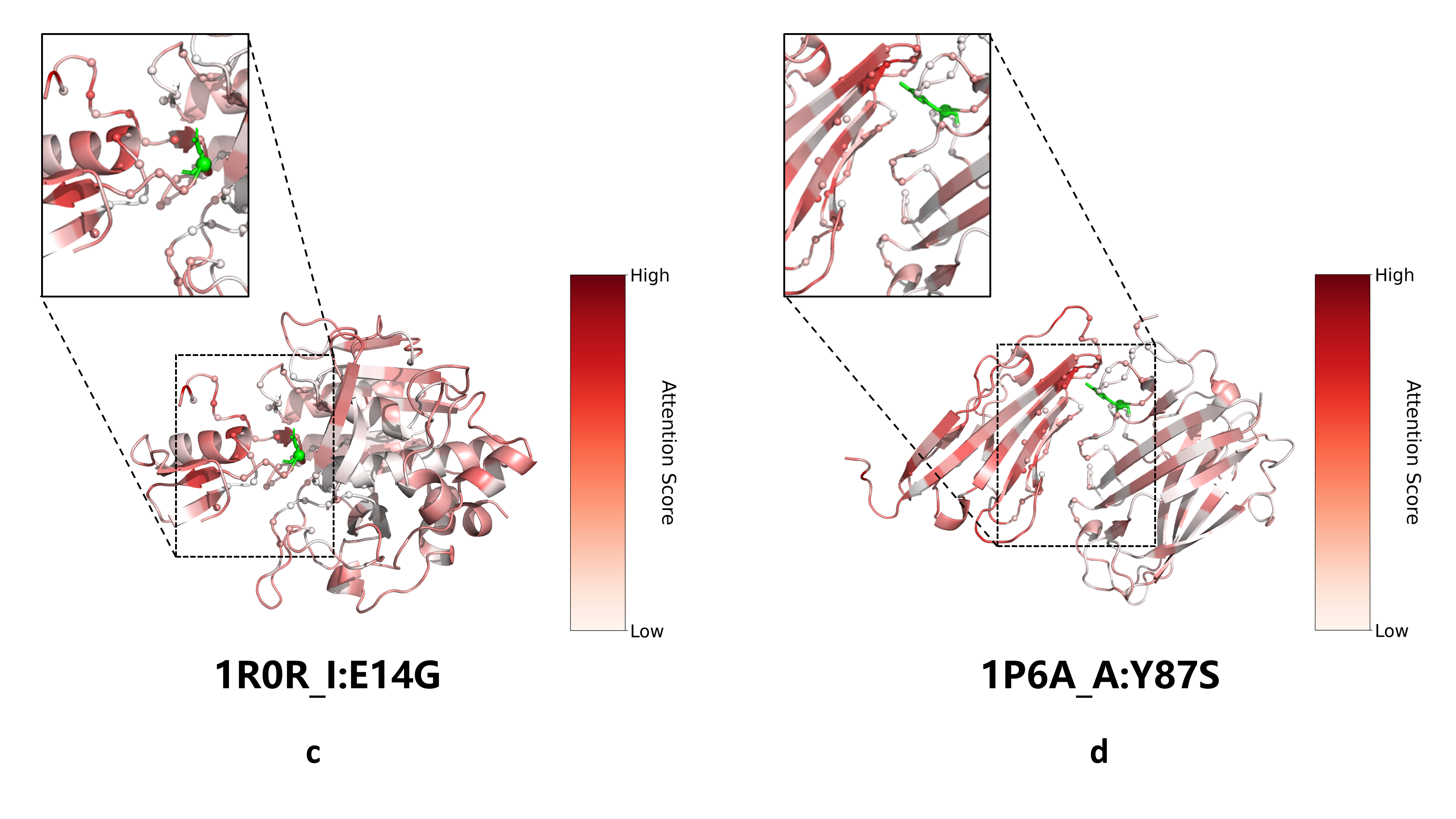


Fig. S2. Illustrative examples of protein structure visualization for contact region analysis. The structure is colored by the attention score, with a deeper red indicating higher attention. Spheres denote contact residues, while the green stick represents the mutation site. Panel a: 1CT0 chain I position 13 Serine mutated to Leucine. Panel b: 1TM1 chain I position 40 Methionine mutated to Phenylalanine. Panel c: 1R0R chain I position 14 Glutamic acid mutated to Glycine. Panel d: 1P6A chain A position 87 Tyrosine mutated to Serine.
